# Decoding endocrine cell differentiation: insights from high‐throughput CRISPR screening in human gut organoids

**DOI:** 10.1002/ctm2.1526

**Published:** 2024-01-27

**Authors:** Lin Lin, Hans Clevers

**Affiliations:** ^1^ Hubrecht Institute Royal Netherlands Academy of Arts and Sciences (KNAW) and UMC Utrecht Utrecht The Netherlands; ^2^ Oncode Institute Utrecht The Netherlands; ^3^ Princess Maxima Center for Pediatric Oncology Utrecht The Netherlands; ^4^ Present address: Pharma, Research and Early Development (pRED) of F. Hoffmann‐La Roche Ltd Basel Switzerland

1

The human gut, our primary digestive organ, has recently garnered recognition as the largest endocrine organ, characterised by its substantial surface area and the abundance of endocrine cells it harbours.^1^ Within the gut epithelium, enteroendocrine cells (EECs) represent a specialized cell type dedicated to endocrine functions. At least a half dozen EEC subtypes are recognized that collectively secrete over 20 distinct hormones, which control feeding behaviour, gut motility, insulin levels and other aspects of metabolism (Figure 1). Notably, the L‐cells, a specific EEC subtype, produce incretin hormones such as glucagon‐like peptide‐1 (GLP‐1) and peptide YY (PYY), both implicated in the regulation of insulin secretion and glucose homeostasis.^2^, ^3^ The clinical significance of EECs is evident in the therapeutic applications of GLP‐1 receptor agonists (GLP‐1RAs) for type 2 diabetes and obesity. To date, nine GLP‐1RAs have received approval from the US Food and Drug Administration (FDA), with numerous others undergoing clinical trials.

**FIGURE 1:**
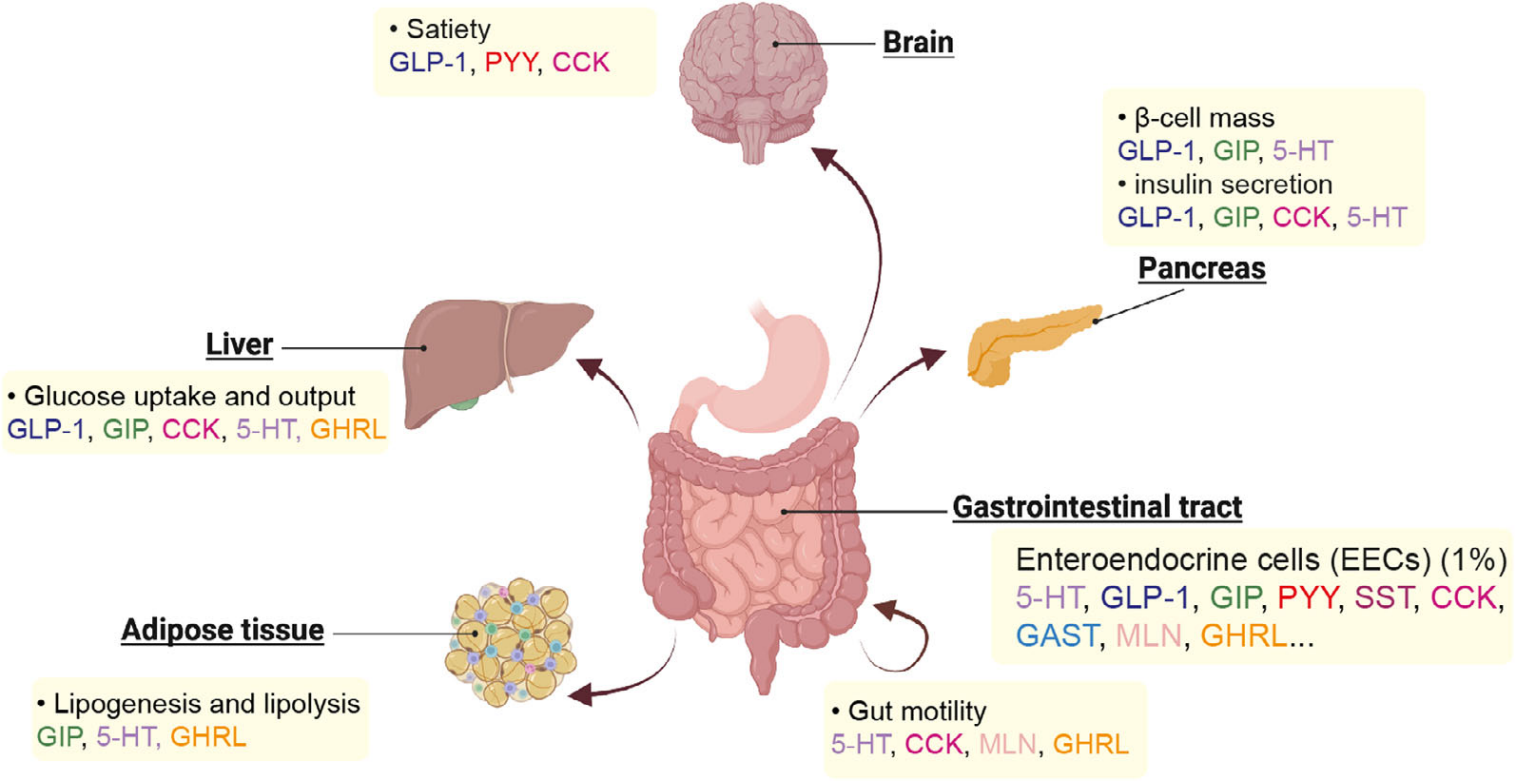
The human gut serves as a significant endocrine organ in the body, with enteroendocrine cells (EECs) constituting approximately 1% of intestinal epithelial cells. These specialised cells play a crucial role by secreting a diverse array of hormones that exert various effects on the body. A few examples are shown here. For instance, in the pancreas, glucagon‐like peptide‐1 (GLP‐1) promotes insulin secretion from pancreatic β‐cells in a glucose‐dependent manner. In the brain, peptide YY (PYY) crosses the blood–brain barrier and decreases hypothalamic neural activation to promote satiety.

Similar to other differentiated cell lineages within the intestinal epithelium, EECs originate from regionally‐specified Lgr5+ intestinal stem cells (ISCs).^4^, ^5^, ^6^ The differentiation of EEC lineages from ISCs is orchestrated by a network of transcription factors (TFs). A prominent player in this regulatory network is *NEUROG3*, recognized as a master regulator of the endocrine cell development in both the pancreas and the gut.^7^, ^8^ By tagging a time‐resolved reporter with mouse *Neurog3*, a real‐time and lineage‐specific map of mouse EEC differentiation at a single‐cell level was constructed.^9^ This study discovered six novel regulators (*Sox4*, *Rfx6*, *Tox3*, *Myt1*, *Runx1t1* and *Zcchc12*) involved in EEC differentiation at different stages. Alternatively, overexpression of NEUROG3 has been used as a tool for generating EECs from ISCs, facilitating in‐depth profiling of human EECs.^10^ Nevertheless, the upstream regulators of NEUROG3 and the downstream effectors of NEUROG3 governing EEC subtype differentiation is not fully explored.

To achieve a more comprehensive understanding of human EEC differentiation, it is crucial to establish an in vitro model faithfully recapitulating the lineage commitment process. The advert of the organoid technology has revolutionized in vitro culture models for biomedical research. In 2009, our group pioneered the intestinal organoid culture, creating the first three‐dimensional self‐organizing tissue in vitro derived from adult stem cells. Intestinal organoids faithfully mimic many aspects of the original tissue physiology, including structural complexity and self‐driven cell fate decisions. Recently, we further refined an optimal culture system for human intestinal organoids, enabling the spontaneous generation of all major differentiated cell lineages, including EECs, without ectopic overexpression of TFs.^11^ This culture model provides a more physiologically relevant in vitro model of human adult endocrine differentiation.

In our recently published study, we integrated the optimal human gut organoid model with the CRISPR‐based genetic screen approach to comprehensively explore the entire repertoire of human TFs involved in EEC differentiation (Figure 2A).^12^ This organoid‐based genetic screening utilised a knockout library comprising 7210 sgRNAs targeting 1800 human TFs, collectively referred to as the TFome. The CRISPR knockout library was first introduced into human small intestinal organoids in the conventional expansion culture by lentiviral transduction. This strategy enabled the identification of 135 essential genes for ISCs, including *KLF5* and *TCF7L2*, whose knockout resulted in impaired cell growth and eventual depletion from the population. Utilising our well‐established differentiation protocol for human intestinal organoids, we enriched EECs from differentiated organoids using the endogenous knock‐in reporter. After assessing the enrichment of sgRNA construct within EECs, we confirmed the essentiality of the TFs (*NEUROG3*, *SOX4*, *INSM1*) classically known to govern EEC differentiation. We further discovered a few TFs (*NFIC*, *TEF*, *ZHX2*) that modulate EEC differentiation, which were not described previously. Key to our study, we identified a previously unstudied TF, *ZNF800*, as a robust repressor of EEC differentiation.

**FIGURE 2:**
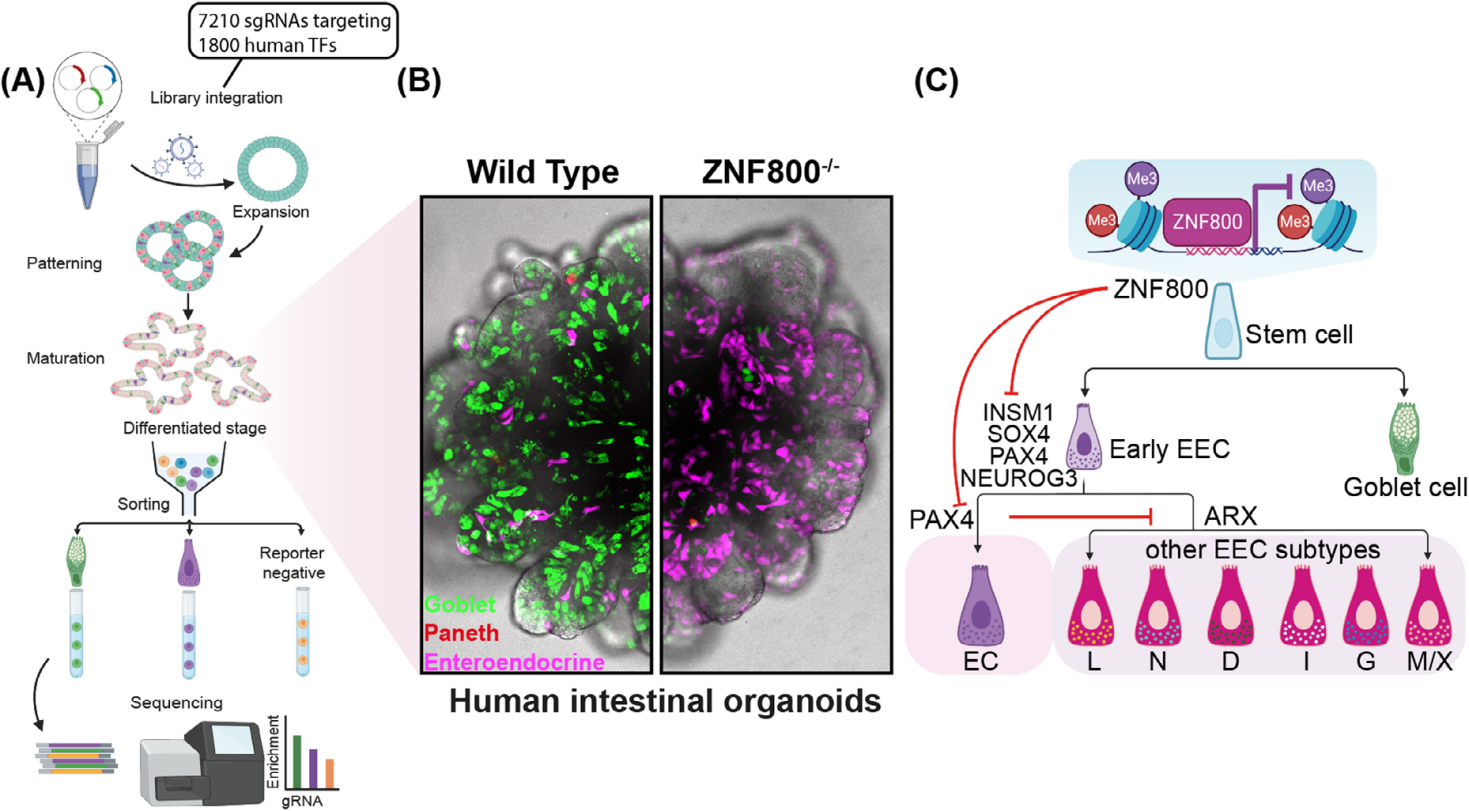
(A) Schematic of CRISPR screen in human intestinal organoids. (B) Representative confocal images of wild type and ZNF800 knockout human small intestinal organoids. Representative marker genes for EECs (CHGA, magenta), goblet (MUC2, green) and Paneth (DEFA5, red) cells. (C) Schematic of the ZNF800‐driven repression model during endocrine cell differentiation.

ZNF800 is a C2H2‐zinc finger protein, which is ubiquitously expressed throughout the body at low levels. In our study, we demonstrate that the knockout of ZNF800 in human intestinal organoids (both ileum and colon) causes pronounced increase in EEC numbers (from ∼1% to ∼15%) (Figure 2B). The robust induction of EECs was accompanied by a significant ablation of other secretory cell types (mainly goblet and Paneth cells). This suggests a shift in the cell fate of ISC differentiation upon ZNF800 loss. Notably, the increased EECs were predominantly identified as enterochromaffin cells (ECs), a specific EEC subtype known for producing serotonin (5‐HT). This finding implies an additional late‐stage shift in the differentiation of EEC progenitors.

Given the limited functional understanding of ZNF800 from previous studies, we conducted a comprehensive analysis of its genome‐wide binding activity using chromatin immunoprecipitation followed by sequencing (ChIP‐seq). This exploration revealed 3085 genes that are bound by ZNF800, exhibiting a gene ontology enrichment in pathways related to neural and the endocrine‐gland development. Notably, key TFs including *NEUROG3*, *PAX4*, *INSM1* and *SOX4* emerged as central players among these downstream targets. Intriguingly, our study demonstrated that only the knockout of PAX4 resulted in the reversal of EEC differentiation, shifting from ECs to other rare EEC subtypes, including L‐cells. Furthermore, we unveiled a unilateral inhibition of PAX4 towards ARX, a TF known to be essential for L‐, G‐, I‐ and N‐cells, indicating PAX4 is responsible for EC lineage specification by suppressing other lineages. Collectively, our downstream analysis provides a mechanistic overview of ZNF800's repressive function in EEC differentiation by directly targeting a network of endocrine TFs (Figure 2C).

Our work highlights the utility of optimized human organoids for CRISPR‐based functional screens, facilitating the identification of regulators in human gut physiology and pathophysiology. While several co‐expression network studies have explored the human endocrine development, ZNF800 has not been discovered through co‐expressed gene modules, primarily due to its ubiquitous expression across the human body. Therefore, functional screening in organoid models demonstrates its potency in high‐throughput target identification, enabling the unbiased interrogation of the gene function in vitro. Moreover, our study provides mechanistic insights in how the endocrine cell development is controlled by both positive and negative regulators. Given the conserved role of *NEUROG3* in the endocrine cell development in both the pancreas and gut, it is a plausible that *ZNF800* may regulate endocrine cell differentiation in the human pancreas through the TF network described in this study. Further investigation in human adult pancreas organoids may address this question.

## AUTHOR CONTRIBUTIONS

**Lin Lin**: Writing – Original Draft Preparation. **Hans Clevers**: Writing – Review & Editing.

## CONFLICT OF INTEREST STATEMENT

H.C. is an inventor on patents held by the Royal Netherlands Academy of Arts and Sciences that cover organoid technology. He is currently head of pharma Research and Early Development (pRED) at Roche, Basel, Switzerland. H.C.’s full disclosure is given at https://www.uu.nl/staff/JCClevers/.

## ETHICS STATEMENT

Not applicable.

## Data Availability

Not applicable.
